# Wild-type isocitrate dehydrogenase under the spotlight in glioblastoma

**DOI:** 10.1038/s41388-021-02056-1

**Published:** 2021-11-11

**Authors:** Gabriel Alzial, Ophelie Renoult, François Paris, Catherine Gratas, Anne Clavreul, Claire Pecqueur

**Affiliations:** 1 Université de Nantes, CRCINA, INSERM, CNRS, F-44000 Nantes, France; 2 Institut de Cancérologie de l’Ouest, Saint-Herblain, France; 3 Université de Nantes, CHU Nantes, Inserm, CRCINA, F-44000 Nantes, France; 4 Université d’Angers, CHU d’Angers, CRCINA, F-49000 Angers, France; 5 Département de Neurochirurgie, CHU Angers, Angers, France

**Keywords:** Biochemistry, CNS cancer, Cell biology, Cancer stem cells

## Abstract

Brain tumors actively reprogram their cellular metabolism to survive and proliferate, thus offering potential therapeutic opportunities. Over the past decade, extensive research has been done on *mutant* IDH enzymes as markers of good prognosis in glioblastoma, a highly aggressive brain tumor in adults with dismal prognosis. Yet, 95% of glioblastoma are IDH *wild-type*. Here, we review current knowledge about IDH *wild-type* enzymes and their putative role in mechanisms driving tumor progression. After a brief overview on tumor metabolic adaptation, we present the diverse metabolic function of IDH enzymes and their roles in glioblastoma initiation, progression and response to treatments. Finally, we will discuss *wild-type* IDH targeting in primary glioblastoma.

## Introduction

GLIOBLASTOMA (GBM) is the most common primary brain tumor in adults, and accounts for more than 2500 cases diagnosed each year in France. This highly malignant and rapidly progressive glioma is distinct histologically from lower-grade tumors by necrosis and hypoxia-induced microvascular hyperplasia. Patients die within 4 months without therapy, while median survival of those receiving radiotherapy with concomitant and adjuvant temozolomide chemotherapy (Stupp protocol) is improved to 15 months [1, 2]. Still, less than 5% of patients survive over 5 years due to invariable GBM relapse [3]. For most patients with GBM, there is no known cause of the disease and no early detection available. Thus, it is essential to better understand the biology of GBM to develop treatment strategies to effectively cure them. One avenue of research that is relatively unexplored in the field of neuro-oncology is how metabolism is rewired in these brain tumors.

Metabolic pathways are core mechanisms that cells use to fuel their growth and survival. One of the major consequences of the genetic and molecular alterations occurring in GBM is an altered cellular metabolism, recognized *in fine* as a key driver of tumor progression. Besides these distinct intrinsic alterations, extrinsic features such as the tumor microenvironment or exposition to treatments may also disrupt activity of several pathways, resulting in distinct metabolic phenotypes [4]. Recent studies have revealed remarkable metabolic heterogeneity and plasticity among GBM but also within distinct regions of the same tumor. In particular, we and others demonstrate that the molecular signature, tumor sublocation in hypoxic region as well as stemness features delineate GBM metabolic rewiring [5–8]. This metabolic heterogeneity would explain why cancer cells with different genetic alterations can display similar metabolic phenotypes whereas cancer cells with identical genetic alterations have different metabolism. One hallmark of metabolic reprogramming is highlighted by enhanced aerobic glycolysis along with excess lactate secretion, termed “the Warburg effect”. Advanced analytical techniques through metabolomics, fluxomic isotope tracers, and metabolic imaging show variations of other critical metabolic circuits including glutaminolysis, one-carbon metabolism, lipid and nucleotide synthesis, as well as reactive oxygen species (ROS) management [9]. While the Warburg effect plays an important role in clinical imaging for cancer through PET scan analysis by measuring higher concentrations of radioactive glucose analog in malignant cancers than in other tissues, this technology is not suitable for GBM due to high background signals. Indeed, the brain is the main consumer of glucose in the body but lacks fuel store, and hence requires a continuously huge supply of glucose [10]. Thus, in the particular context of GBM, development of novel radiotracers based on amino acid or lipid metabolism would definitively improve GBM diagnosis and follow-up, which are currently mainly resting on common imaging methodologies such as MRI and CT scans. Furthermore, therapeutic opportunities might arise if we can identify specific metabolic liabilities in GBM cells, distinct from canonical metabolic pathways supporting cell growth of normal cells.

Recent insights in metabolomic studies have suggested a key role of *wild-type* IDH enzymes upon treatment to favor GBM proliferation and recurrence [11]. The discovery that patients with *mutant* IDH1/2 GBM have a better outcome compared to those with *wild-type* enzymes has spurred robust research to study the consequences of IDH mutations on cellular metabolism and to design new effective targeted molecular therapies. Given that *wild-type* isoforms account for more than 90% of all GBM, it is now time to capitalize on the knowledge built from *mutant* IDH1/2 targeting to outline rationale on *wild-type* IDH enzymes targeting. Here, we provide an overview of the metabolic properties of IDH enzymes and their potential as new therapeutic targets against GBM.

## Metabolic properties of IDH Enzymes

**Fig. 1:**
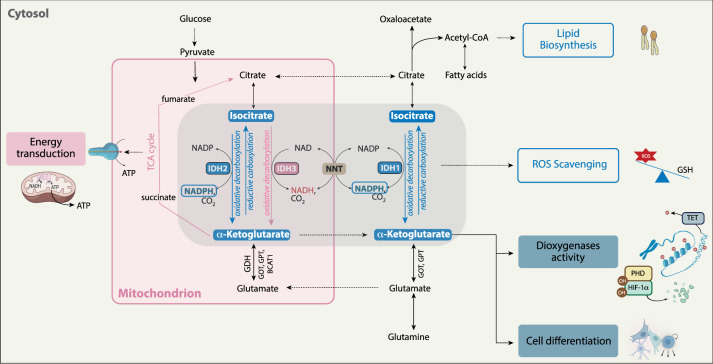
Metabolic properties of *wild-type* IDH enzymes. Depending on the isoform, the cofactor, and the localization, IDH enzymes are involved in different cellular processes including mitochondrial energy production, glutamine metabolism, lipogenesis, epigenetic profile, cell responses to hypoxia and cellular redox status. IDH1 performs its function in the cytosol, while IDH2 and IDH3 function as part of the tricarboxylic acid (TCA) cycle in the mitochondria. All three IDH isoforms catalyze the oxidative decarboxylation of isocitrate to α-ketoglutarate and carbon dioxide with the production of reducing equivalent NAD(P)H. Whereas this reaction is irreversible through IDH3 within the TCA cycle, IDH1/2 activities are working in a reversible manner.

IDH enzymes have been known for decades to catalyze the oxidative decarboxylation of isocitrate producing alpha-ketoglutarate (αKG) and carbon dioxide (CO_2_) while reducing cofactors NAD(P)^+^ to NAD(P)H (Fig. 1). In all eukaryotic cell types except mature red blood cells, three different IDH paralogs exist. IDH1 and IDH2 are homodimeric NADP^+^-dependent enzymes and mostly differed by their localization, IDH1 being cytoplasmic while IDH2 is expressed in mitochondria. In contrast, IDH3, also expressed in the mitochondria, uses NAD^+^ as cofactor, forms heterodimers or heterotetramers composed of αβ and αγ subunits and works in an irreversible manner. These 3 IDH isoforms have overlapping but nonredundant roles in metabolism including, but not limited to, mitochondrial oxidative phosphorylation, glutamine metabolism, lipogenesis, glucose sensing, and regulation of cellular redox status [12, 13] (Fig. 1*)*.

### Canonical functions of IDH enzymes

#### **Production of αKG, a mitochondrial key metabolite with pleiotropic activity**

Mitochondrial metabolites generated through the tricarboxylic acid (TCA) cycle are crucial for the biosynthesis of macromolecules such as nucleotides, lipids, and proteins. TCA cycle is mainly fueled through 2 metabolic inputs, one from glucose-derived pyruvate and the other one from glutamine-derived αKG through the glutamate dehydrogenase (GDH). While αKG is produced by all 3 IDHs by the oxidative decarboxylation of isocitrate, IDH3 is the main producer of αKG within the TCA cycle (Fig. 1*)*. This oxidative decarboxylation catalyzed by IDH3 is irreversible and tightly regulated. Indeed, IDH3 activity is allosterically regulated by substrate availability, product inhibition, and the cell redox status to avoid unnecessary depletion of isocitrate and accumulation of αKG [14]. Once in the TCA cycle, αKG is further metabolized to succinate then fumarate through the succinate dehydrogenase and the fumarate hydratase, respectively. In combination with IDH3, IDH2 regulates the TCA cycle running through its ability to work in a reversible manner by converting αKG back to isocitrate (Fig. 1*)*. This cycle is completed by the transfer of electrons from NADH to NADPH through the nicotinamide nucleotide transhydrogenase [15]. Of note, other enzymes, including glutamate-pyruvate transaminases (GPT1/2) and glutamate-oxaloacetate transaminase (GOT1/2), can also produce αKG, allowing parallel synthesis of alanine and aspartate, respectively, that can also be used as precursors for TCA cycle intermediates and protein synthesis.

Besides its critical role in metabolic cellular homeostasis, αKG is also an obligatory cofactor of dioxygenase enzymes, a large group of phylogenetically conserved enzymes including the prolyl-hydroxylase (PHD) and multiple demethylases, which play a key role in important processes such as responses to hypoxia and chromatin modifications respectively. Precisely, αKG regulates PHD activity involved in the stabilization of the hypoxia-inducible factor-1α (HIF-1α), a master regulator of oxygen homeostasis (Fig. 1*)*. Under limited oxygen conditions or reduced levels of αKG, PHD activity is impaired resulting in HIF-1α translocation to the nucleus where it regulates the transcription of genes mainly involved in metabolism, erythropoiesis, and angiogenesis, as well as stem and immune cell function [16]. Importantly, while supraphysiological concentrations of TCA intermediates succinate and fumarate can inhibit PHD under normoxia, increased intracellular αKG can reactivate PHD in hypoxic cells resulting in metabolic catastrophe and cell death [17]. Alpha-KG is also required for the activity of some demethylases involved in controlling chromatin modifications and DNA methylation including the ten-eleven translocation (TET) DNA hydroxylases and the Jumonji histone demethylases (Fig. 1*)*. Since histone and DNA methylation have a direct impact on gene transcription, the available pool of αKG modulates cell fate decision. For example, embryonic stem cells exhibit a high level of intracellular αKG to promote histone and DNA demethylation and maintain stem cell self-renewal and pluripotency [18].

#### **Formation of reducing equivalents involved in ATP production, lipid synthesis, and antioxidant defense**s

Besides αKG production, the oxidative decarboxylation catalyzed by the 3 IDH isoforms leads to the formation of reducing equivalents, NAD(P)H. IDH3 activity directly generates NADH production as well as FADH2 by promoting TCA cycle running. These reducing equivalents are used by the electron transport chain (ETC) to produce ATP. In contrast, IDH1/2 leads to the formation of NADPH, a key molecule involved in lipid synthesis and the antioxidant machinery (Fig. 1*)*.

Fatty acid and lipid biosynthesis reactions are major users of NADPH. For example, the synthesis of one palmitate (16:0) from acetyl-CoA and malonyl-CoA by fatty acid synthase requires the input of 14 molecules of NADPH. Although the association of NADPH production and lipogenesis is well known, direct evidence of IDH1/2 involvement has been demonstrated only recently. Transgenic mice overexpressing IDH1 in the liver and adipose tissues experienced obesity and hyperlipidemia, paralleled by increased triglyceride and cholesterol content [19]. Conversely, in vivo IDH1 invalidation resulted in weight loss associated with reduced fat mass and circulating triglycerides levels [20]. In the brain, IDH1 has been shown to regulate phospholipid metabolism in developing astrocytes [21].

Reducing equivalents supplied by NADPH also secure an adequate pool of reduced glutathione (GSH) and thioredoxin to protect the cell from ROS that cause DNA damage, protein oxidation, and lipid peroxidation [22]. The role of IDH1 and IDH2 as protectors against various insults has been confirmed extensively by several groups. Notably, Lee et al. have shown that IDH1 or IDH2 deficiency in mouse embryonic fibroblasts leads to increased lipid peroxidation, oxidative DNA damage, intracellular peroxide generation, and decreased survival after oxidative stress, while overexpression of either IDH1 or IDH2 prevents these effects [23, 24].

### Reductive carboxylation as a metabolic adaptation of mitochondrial impairment

The reductive carboxylation is the reverse reaction of the oxidative decarboxylation and can be exclusively catalyzed through IDH1 and IDH2 enzymes using glutamine-derived αKG to produce isocitrate along with NADP+ (Fig. 1*)*. As seen above, pools of reducing equivalents are regulated through an isocitrate/αKG cycle where the irreversible oxidative carboxylation catalyzed by IDH3 is coupled to the reductive decarboxylation catalyzed by IDH2.

Several recent publications revealed the importance of this glutamine-dependent reductive carboxylation for *de novo* lipogenesis in cells exhibiting mitochondrial dysfunction or upon hypoxia [5, 25, 26]. This reaction allows citrate formation, without passing through the conventional clockwise steps of the TCA cycle, to produce acetyl-CoA and fuel *de novo* fatty acid biosynthesis, that are key membrane components and important signal transducers. Of note, glutamine-dependent reductive carboxylation has been previously described as a minor source of isocitrate/citrate and lipogenic carbon in a restricted number of normal cells from liver, heart, brown adipocytes, retinal pigment epithelium, and quiescent fibroblasts [27–31]. While αKG/citrate ratio is a critical determinant of glutamine-dependent reductive carboxylation [32], this reaction is inhibited by NADP^+^ and, to a lesser extent, by isocitrate [33]. Thus, reductive carboxylation retains glutamine as a crucial growth-promoting nutrient when mitochondrial metabolism is impaired.

## IDH enzymes as crucial players in GBM

**Fig. 2:**
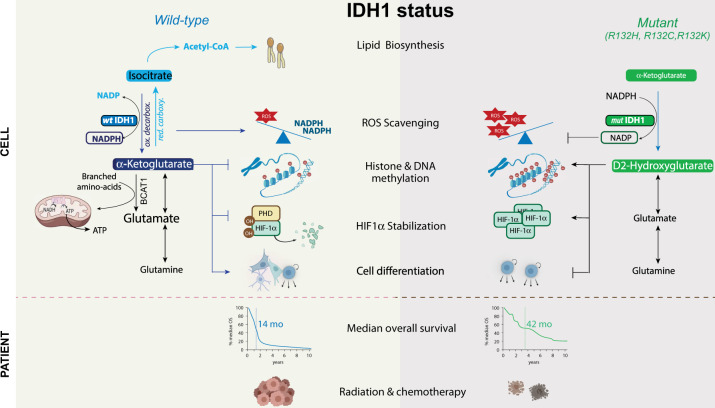
Metabolic discrepancies between *wild-type* and *mutant* IDH1 in GBM. Hotspot mutation in IDH1 gene has been identified in GBM occuring at the active site within the catalytic pocket, and resulting in a neomorphic activity leading to the generation of (D)2-Hydroxyglutarate (D2HG) while oxidizing NADPH. D2HG, through structural similarity to αKG, acts as a competitive inhibitor leading to inhibition of αKG-dependent dioxygenases, and resulting to epigenetic alteration, HIF1α stabilization, and alterations in cellular differentiation and response to oxidative stress. Tumors with IDH1/2 mutations have distinctive genetic and clinical characteristics. In particular, patients with *mutant* IDH1/2 GBM have a better outcome compared to those with *wild-type* IDH tumor.

In 2008, hotspot mutation in IDH1 gene was identified in grade II/III astrocytomas and oligodendrogliomas, and in secondary GBM that developed from these lower-grade lesions [34, 35]. Secondary GBM without IDH1 mutation often had mutations on the IDH2 gene. This was rapidly followed by identification of recurrent IDH1/2 mutations in other tumor types, including acute myeloid leukemia (AML). GBM with IDH mutations are clinically and genetically distinct from GBM with *wild-type* IDH genes. In particular, patients with *mutant* IDH1/2 GBM have a better outcome compared to those with *wild-type* IDH tumor (14 months with *wild-type* IDH vs 42 months with *mutant* IDH) (Fig. 2*)* [36]. Mutant IDH tumors are also associated with extensive epigenomic alterations revealed by a global hypermethylation landscape (G-CIMP phenotype). These particular characteristics prompted the World Health Organization (WHO) in 2021 to refer *mutant* IDH GBM as grade 4 mutated IDH astrocytoma, to distinguish more clearly between this entity and *wild-type* IDH GBM [37]. Thus, while IDH enzymes have been known for decades, their contribution to GBM aggressiveness and recurrence has been barely studied until the identification of their mutations. The emerging literature showing how the metabolic functions of IDH enzymes impact tumor initiation, progression, dissemination, and treatment escape in GBM is presented below (Fig. 2*)*.

### Metabolic functions of *wild-type* IDH1 in GBM

Recent studies have highlighted the importance of *wild-type* IDH1 in GBM progression. First, Calvert et al. reported that *wild-type* IDH1 is overexpressed in most primary GBM [38]. Notably, in GBM samples specimens profiled by The Cancer Genome Atlas Consortium, IDH1 appeared as the most differentially expressed NADPH-producing enzyme compared to normal brain tissue [38, 39] and exhibited a higher maximal enzymatic activity than other NADPH-producing enzymes in patient-derived GBM samples [40]. Through its oxidative decarboxylation activity, IDH1 promotes tumor progression and resistance to cell death through efficient fatty acid synthesis and ROS scavenging activities (Fig. 2*)* [41]. Accordingly, its genetic or pharmacological inhibition reduced tumor growth, both in vitro and in vivo. Furthermore, and in agreement with its ROS scavenging activity, upregulation of IDH1 expression was observed following ionizing radiation and its silencing increased tumor sensitivity to radiation-induced senescence, both in vitro and in murine xenograft models of human GBM [39]. Finally, rescuing IDH1 metabolic activities was sufficient to reverse this process.

The reductive carboxylation activity of *wild-type* IDH1 also plays a crucial role in tumor cells located in hypoxic regions, which are frequently found in GBM and have been associated with tumor aggressiveness, invasion, and resistance to therapies. Upon hypoxia, tumor cells rely almost exclusively on glutamine-dependent reductive carboxylation catalyzed by IDH1 for lipids synthesis while, in normoxia, lipids are preferentially synthesized from glucose [5, 25, 42]. Accordingly, knockdown of IDH1 reduced glutamine-dependent reductive carboxylation and impaired cell proliferation, under hypoxia [25].

IDH1-derived αKG can also be transaminated to glutamate through the branched-chain amino acid transaminase-1 (BCAT1). In the brain, glutamate plays a crucial role as a neurotransmitter and also presents clinical relevance in GBM. Indeed, several studies have reported that increased level of glutamate promotes both tumor progression and invasion by providing macromolecule precursors and reducing equivalents for mitochondrial ATP synthesis as well as increasing antioxidant production mainly through GSH synthesis (Fig. 2*)* [43, 44]. Interestingly, cytoplasmic BCAT1 has been shown to be significantly upregulated in GBM expressing *wild-type* IDH1 while not being expressed in GBM expressing *mutant* IDH1 implying a mechanistic link between these two enzymes [45]. This hypothesis was reinforced by the decreased BCAT1 expression in GBM cells upon IDH1 silencing. Importantly, deregulation of either branched-chain amino acid metabolism and glutamate secretion result in neuronal dysfunction and excitotoxic death [46]. Thus, blocking this metabolism should reduce tumor growth by altering tumor energy production and macromolecules synthesis, as well as limit peritumoral seizures experienced by GBM patients early in the disease.

### Metabolic functions of *wild-type* IDH2 and IDH3 in GBM

**Fig. 3:**
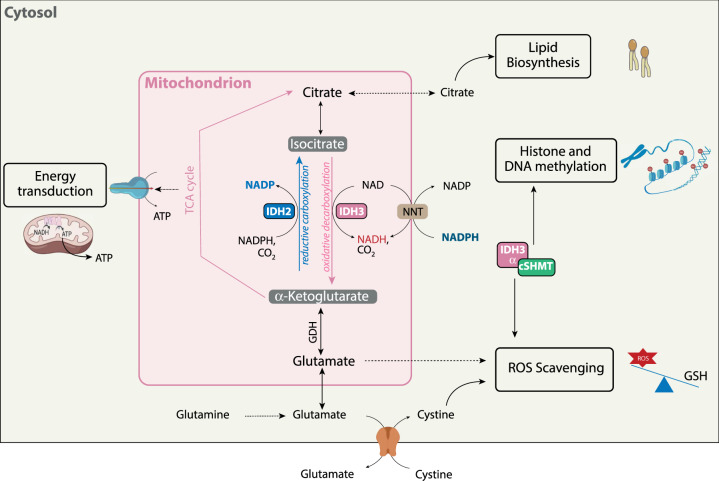
Metabolic functions of *wild-type* IDH2 and IDH3 in GBM. IDH2 and IDH3 are located in the mitochondria with the respective production of NADP(H) and NADH. These 2 isoforms act in concert to regulate energy production through modulation of TCA cycle running through an isocitrate/αKG cycle. In this cycle, IDH3 converts isocitrate to αKG while IDH2 converts αKG and NADPH back to isocitrate and NADP^+^. This metabolic cycle allows tumor cells to favor lipid biosynthesis and to cope with mitochondrial oxidative stress. In mitochondria, αKG is provided by the glutamate dehydrogenase (GDH) from glutamine through glutamate. The subunit IDH3α can be found in the cytosol where it interacts with serine hydroxymethyltransferase (cSHMT), an enzyme involved in epigenetic profiling through histone and DNA methylation.

Few studies have been performed to study the role of *wild-type* IDH2 and IDH3 in GBM. As seen above, these two mitochondrial isoforms act in concert to regulate TCA cycle running through an isocitrate/αKG cycle where IDH2 mainly converts αKG and NADPH to isocitrate and NADP^+^ while IDH3 converts isocitrate back to αKG (Fig. 3*)*. This cycle is regulated by substrate availability, product inhibition, and cell redox status [14]. In particular, IDH2 reductive carboxylation is increased in highly glycolytic cells or cells with dysfunctional ETC [47, 48]. In contrast, while an excess of NAD + over NADH leads to IDH3-dependent oxidative decarboxylation, an increased concentration of NADH and a shortage of NAD + reroute αKG to glutamate through GDH This IDH2/IDH3 metabolic cycle also allows tumor cells to cope with mitochondrial oxidative stress generated by the disruption of the respiratory chain or induced by chemo- or radiotherapy [26, 48, 49].

Besides their combined regulation of the TCA cycle running, each isoform also provides individual metabolic benefits to tumor cells. Indeed, IDH2-dependent reductive carboxylation is required for cell survival and proliferation under hypoxia or in absence of glutamine as shown by the inability of IDH2-deficient GBM cells to proliferate in such conditions [5]. In contrast, IDH3, through interaction of its subunit IDH3α with the cytosolic serine hydroxymethyltransferase (cSHMT), enhanced both nucleotide availability and DNA methylation favoring GBM progression in murine orthotopic GBM models [50]. Indeed, May et al. have recently demonstrated that IDH3α, which was predominantly expressed in mitochondria, can also be detected in the cytosol where it binds to cSHMT. This enzyme controls a rate-limited step in one-carbon metabolism, a central metabolic pathway that uses folate to support nucleotide synthesis, DNA and protein methylation as well as *de novo* thymidine and purine synthesis pathway (Fig. 3) [50, 51]. Importantly, they also reported that IDH3α was overexpressed in GBM patient samples [50].

### Metabolic functions of *mutant* IDH1/2 in GBM

IDH1/2 mutations are exclusively heterozygous and result in one amino acid change, at residue R132 in IDH1 and R140 or R172 in IDH2, located at the active site within the catalytic pocket. Mutant IDH1/2 are unable to carry out *wild-type* IDH activities. Rather, these mutants catalyze the NADPH-dependent reduction of αKG to the oncometabolite (D)2-Hydroxyglutarate (D2HG) (Fig. 2*)* [52, 53]. This oncometabolite is not present in normal cells but accumulates considerably in tumors with *mutant* IDH. In line with its neomorphic activity, *mutant* IDH1 GBM engrafted in mouse brain display high levels of D2HG and exhibit very slow tumor growth [54].

At the cellular level, through its structural similarity to αKG, D2HG inhibits αKG-dependent dioxygenases leading to epigenetic changes [55–57], HIF1α stabilization [58], and alterations in cellular differentiation status [59]. In fact, accumulation of D2HG in tumor cells is sufficient to establish the global hypermethylation landscape characteristic of *mutant* IDH1/2 GBM [57]. Mutation in IDH1 also triggers a bioenergetic metabolic switch in GBM cells leading to a significant increase in oxidative mitochondrial metabolism for the generation of ATP through both an increase in the number of mitochondria and the utilization of glutamate and pyruvate (for review see [60]). IDH1/2 mutation also imposes a shortage of the reducing equivalents required to ensure antioxidant functions. Further in vitro studies demonstrated that *mutant* IDH1/2 alters the TCA metabolic fluxes leading to increased dependence on glutaminolysis [61–63] and compromised multiple DNA repair pathways, ultimately making tumor cells more susceptible to radiation and chemotherapy [64–67].

Hence, both *mutant* and *wild-type* IDH enzymes may constitute a cornerstone of tumor aggressiveness and dynamic metabolic plasticity, in primary and secondary GBM, allowing tumor cells to cope with multifactorial stresses.

## Targeting of wild-type IDH enzymes in GBM

Given the dismal prognosis of GBM, new therapeutic approaches are urgently required. Recent identification of neomorphic IDH1/2 mutations in secondary GBM has generated robust research to elucidate their role in gliomagenesis, tumor progression and impact on clinical outcome. Several small molecules that directly inhibit mutant IDH1/2 activities have been developed, with some of them currently evaluated in phase I/II/III clinical studies in secondary GBM (Table 1) (for detailed review see [68]). However, IDH-targeting therapeutic approaches are currently restricted to *mutant* IDH1/2 GBM while they represent less than 10% of highly malignant glioma. According to their involvement in a wide range of metabolic processes, *wild-type* IDH-mediated metabolic reprogramming could also be a key driver of tumor adaptation allowing GBM proliferation, tumor escape, and recurrence. Thus, further investigations should build on *mutant* IDH1/2 knowledge to propose new therapeutic approaches targeting *wild-type* isoforms. Here, we will give a brief overview of *mutant* IDH1/2 inhibitors and discuss *wild-type* IDH targeting in primary GBM.

**Table 1:** Genetic and pharmacological inhibition of *wild-type* IDH enzymes.

Therapeutic agents	Target	Glioma models	Cellular responses	IC50 IDHmut	IC50 IDHWT	References	Clinical trials (phase)
Direct chemical inhibitors							
*Ivosidenib (AGI-120)*	IDH1^mut^	Human U87MG cell line		12 nM	71 nM	[95]	NCT02073994 (I) NCT03343197 (I) NCT04056910 (II) NCT02989857 (III)
*Vorasidenib (AGI-881)*	IDH1^mut^/IDH2^mut^	Human GBM cell lines U87MG and TS603)/Murine xenograft of TS603	↘ 2-HG production in vivo	0.25–7 nM	n.d.	[78]	NCT02481154 (I) NCT03343197 (I) NCT04164901 (III)
*Enasidenib (AGI-221)*	IDH2^mut^			n.d.	n.d.		NCT02273739 (I-II)
*AGI-5198*	IDH1^mut^	Human U87MG cell line/Murine xenograft of U87MG Human TS603 GBM cell line/Murine xenograft of TS603	↘ 2-HG production and tumor growth in vivo ↘ histone methylation, ↗ astroglial differentiation, ↘ 2-HG production and tumor growth in vivo	70 nM	70 nM	[72, 73]	
*AGI-6780*	IDH2^mut^ IDH2^WT^	Human U87MG cell line		11 nM	190 nM	[96]	
*BAY-1436032*	IDH1^mut^ IDH1^WT^	Human glioma cell line (LN299)/patient-derived secondary GBM	↘ astrocytome proliferation, ↗ cell differentiation ↗ mice survival	47 nM	20 µM (cell-free assay)	[97]	NCT02746081 (I)
*FT-2102*	IDH1^mut^	Human U87MG cell line		9 nM	24 µM (cell-free assay)	[98]	NCT03684811 (I-II) 2018-001796-21 (I)
*IDH-305*	IDH1^mut^			n.d.	n.d.		NCT02381886 (I)
*GSK864*	IDH1 ^WT^	Patient-derived glioma initiating cells/PDXs	↘ tumor proliferation ↗ mice survival, ↗ RTK inhibitor efficacy	n.d.	n.d.	[38]	
Immunotherapy							
*Peptide vaccine*	IDH1^mut^	Murine GL261 GBM cell line/murine graft of GL261	↗ anti-tumor immune response, ↗ mice survival	n.d.	n.d.	[80]	NCT02454634 (I) NCT03893903 (I)
SiRNA/ShRNA therapeutics							
*ShRNA*	IDH1^WT^	Patient-derived glioma initiating cells/PDXs	↘NADPH and α-KG levels, ↗ cell differentiation and ROS level, ↘ stemness and tumor proliferation, ↗ mice survival	n.d.	n.d.	[38]	
*Si/ShRNA*	IDH1^WT^	Human GBM cell lines (U87MG, A172 and U138MG)/ Murine xenograft of U87MG	↘ deoxynucleotide and antioxidant pools ↘ tumor proliferation in combination with irradiation in vivo	n.d.	n.d.	[39]	
*SiRNA*	IDH2^WT^	Human SF188 GBM cell line	↘ cell proliferation and reductive carboxylation in hypoxia	n.d.	n.d.	[5]	
*CRISPR/Cas9 silencing*	IDH3^WT^	Patient-derived glioma initiating cells	↘ NADPH/NADP^+^ and nucleotides synthesis. ↘ tumor proliferation ↗ epigenome methylation, methotrexane sensitivity.	n.d.	n.d.	[50]	

Several studies have been performed to inhibit *wild-type* IDH enzymes. Small molecules that directly inhibit *mutant* IDH1/2 activities have been tested against *wild-type* IDH enzymes as well as genetic inhibition through RNA interference. n.d. undetermined

### Lessons from IDH1/2 mutations in secondary GBM

One striking difference between *wild-type* IDH GBM and *mutant* IDH1/2 tumors resides in their methylation landscape, which is known to play important roles during oncogenesis [57] (Fig. 2). Thus, initial preclinical studies used pan-methylases inhibitors such as the FDA-approved drugs 5-azacitidine (5-aza) and decitabine. Treatment of *mutant* IDH1 GBM bearing mice with either agent resulted in a dramatic loss of stem-like properties and decreased tumor growth [69–71]. However, their impact on the epigenomic landscape of normal cells strongly limits their clinical applications. Furthermore, while DNA hypermethylation elicits tumorigenesis through silencing of tumor suppressor gene, DNA hypomethylation also contributes to oncogenesis through induction of genomic instability and oncogene activation. Since D2HG is sufficient to establish *mutant* IDH1/2 GBM hypermethylation phenotype, several compounds that directly inhibit *mutant* IDH1/2 enzymes have been developed with promising results (Table 1). Independently of the targeted mutant isoform, most of them reduced D2HG production in vitro and are able to penetrate the blood–brain barrier. In orthotopic mouse models of *mutant* IDH1 GBM, their oral administration reduces intratumoral D2HG, reverse histone and DNA hypermethylation, and prolong mice survival [72–74]. Based on these preclinical evidence, several clinical trials are currently ongoing on *mutant* IDH1/2 glioma, including GBM, to evaluate the safety and efficacy of *mutant* IDH1 inhibitors (Ivosidenib, BAY1436032 and IDH305), *mutant* IDH2 inhibitor (Enasidenib), and pan-inhibitors inhibiting both mutant isoforms (Vorasidenib) [75–78].

Recently, innovative *mutant* IDH1 targeting has been published exploiting R132H mutation as a cancer-specific epitope to design protein-specific vaccine [79]. In preclinical syngeneic models, peptide vaccination increased survival of mice bearing *mutant* IDH1 GBM through CD8+T cell response, specific cytotoxicity, and an antibody response [80]. A recent phase I trial was carried out in 33 patients with newly diagnosed grade 3 and 4 *mutant* IDH1 astrocytomas to evaluate the safety and tolerability, as well as immune responses to the peptide vaccine (NOA-16) [81]. NOA-16 demonstrated safety and immunogenicity in 93.3% of patients across multiple MHC alleles. These results are encouraging but the high frequency of pseudoprogression, which was associated with increased vaccine-induced peripheral T cell responses, need further functional investigations using trial tissues.

### Genetic and pharmacological targeting of *wild-type* IDHs

In agreement with the crucial role of IDH1 in anti-oxidant defenses through NADPH production, recent studies have demonstrated that its genetic inhibition reduces GBM growth and may significantly improve the efficacy of conventional GBM therapies [38, 39] (Table 1). Indeed, inactivation of IDH1 through RNA interference reduces GBM growth and prolongs the survival of mice bearing patient-derived xenografts. These effects were mediated through inhibition of the oxidative decarboxylation of isocitrate to αKG resulting at the molecular level to impaired lipid and deoxynucleotide biosynthesis and increased ROS production, due to reduced levels of αKG and NADPH. These molecular alterations also resulted in increased tumor cell sensitivity to both radiation-induced senescence and erlotinib-induced apoptosis [38, 39]. Indeed, increased ROS production combined with reduced NADPH and deoxynucleotide pools trigger GSH exhaustion and increase double-strand DNA breaks leading to cell death. One study also reported that *wild-type* IDH1 silencing significantly reduced the frequency of GBM stem-*like* cells involved in GBM recurrence [38]. Importantly, they also demonstrated that pharmacological inhibition of *wild-type* IDH1 recapitulates its genetic silencing [38]. Indeed, GSK864, a compound initially identified as a potent inhibitor of mutant IDH1 in AML [82], inhibits *wild-type* IDH1 activity, reduces GBM stem-*like* cell frequency and increases survival of tumor-bearing mice. In contrast to IDH1, no significant metabolic change was observed after IDH2 silencing by RNA interference under normoxia [25]. These results are in agreement with a crucial role of IDH2 in particular conditions such as hypoxia [5].

Genetic inhibition of IDH3α in orthotopic GBM mouse models also decreases cell growth through accumulation of pyrimidine pathway intermediates, increase of total NADPH/NADP + ratio and altered DNA methylation profile [50]. These epigenetic alterations induced by IDH3α deletion deregulate key pathways such as cyclic adenosine 3′, 5′-monophosphate-mediated signaling and epithelial-to-mesenchymal transition. Hence, blunted nucleotide biosynthesis, together with epigenetic silencing of potent growth and multipotency factors in response to IDH3α loss of function, creates a unique metabolic vulnerability in highly proliferative GBM cells, that decreases cellular viability. Furthermore, IDH3α extinction cooperates with antifolate therapy, such as methotrexate (MTX), known to target the thymidylate pathway enzymes DHFR and TYMS, to promote programmed cell death [50].

### Future strategies to target *wild-type* IDH enzymes

Published data indicate that *mutant* as well as *wild-type* enzymes, are interesting actionable therapeutic targets. Unfortunately, whereas *mutant* IDH1 inhibitors have been developed, they cannot be directly used in *wild-type* IDH GBM. First, while some compounds, such as ivosidenib, AGI-6780, and BAY-1436032, may also inhibit *wild-type* IDH1 activity, required doses are usually too high to be further evaluated in clinics (Table 1). Second, while *mutant* IDH enzymes display one unique and specific neomorphic activity, *wild-type* IDH enzymes catalyze several metabolic reactions involved in different cellular processes depending on their intracellular sublocation and microenvironment. Third, GBM being highly heterogeneous, other factors such as their wider mutational profile, including P53, PTEN, or EGFR, as well as their molecular signature or their anti-oxidant profiles, may alter *wild-type* IDH metabolic functions [83]. Finally, a recent computational analysis identified four stable tumor cell states with divergent mitochondrial glucose, glutamine, and lipid metabolism, in addition to specific neurodevelopmental features and different patient outcomes [84]. In particular, the mitochondrial subset of GBM cells relies exclusively on oxidative phosphorylation for energy production, in contrast to glycolytic/plurimetabolic subset sustained by activation of multiple energy-production programs including aerobic glycolysis, amino acids, and lipid metabolism. Thus, the identification of key *wild-type* IDH-mediated metabolic activity, depending on the genetic and metabolic landscape and involved in GBM aggressiveness, is a prerequisite for further development of specific *wild-type* IDH inhibitors in preclinical and clinical studies. The canonical function of IDH enzymes, namely the oxidative decarboxylation of isocitrate to αKG, is hardly targetable since it is displayed by most cells, both normal and tumoral. However, the reductive carboxylation catalyzed by IDH1 and IDH2 only occurred in anchorage-independent tumor cells, cells with altered mitochondria, or located in hypoxic niches [25, 42, 85]. Accordingly, glutamine-derived reductive carboxylation was barely detected in normoxia and was not affected by IDH1 or IDH2 silencing [25]. In contrast, IDH2-mediated reductive carboxylation becomes critical for tumor proliferation upon hypoxia [5]. This is of particular interest since the most aggressive GBM cells, including GBM stem-*like* cells and mesenchymal GBM cells, have been shown to reside in hypoxic niches [86–89]. GBM stem-*like* cells display self-renewal ability and long-term proliferation, potent tumor initiation ability, and radio- and chemo-resistance [90–92]. Mesenchymal GBM cells are predominantly present in *wild-type* IDH GBM, are associated with poor radiation response and worse survival [93]. Importantly, global molecular signatures of most GBM relapses are mesenchymal [94]. Thus, targeting IDH-mediated reductive carboxylation may be a potent way to efficiently eradicate these highly malignant cells while sparing normal cells.

## Conclusion

In conclusion, *wild-type* IDH enzymes appear as potent actionable therapeutic target in order to improve primary GBM prognosis. A therapeutic strategy of targeting IDH enzymes via small molecules in combination with targeted and/or conventional therapies could represent a Gordian knot solution and may meet more success than solely targeting genomic alterations in a heterogeneous tumor such as GBM. Importantly, several studies targeting *wild-type* IDH enhances GBM responsiveness to treatments and provides a strong rationale to develop IDH targeted therapies. Finally, since cancers upregulate a variety of metabolic genes that conspire to reprogram tumor cell metabolism, support intense growth and therapy resistance, a deep investigation of all potential metabolic pathway inhibition in combination, or not with other therapies, should hopefully lead to therapeutic advances that will improve the dismal outcomes currently seen for GBM patients.
